# Online Learning Communities and Mental Health Literacy for Preschool Teachers: The Moderating Role of Enthusiasm for Engagement

**DOI:** 10.3390/ijerph16224448

**Published:** 2019-11-13

**Authors:** Pi-Chun Hsu, I-Hsiung Chang, Ru-Si Chen

**Affiliations:** 1 School of Education Science, Minnan Normal University, Zhangzhou 363000, China; lilian119119@gmail.com; 2 Department of Early Childhood Education, TOKO University, Puzi 61363, Taiwan; elite5931.tw@gmail.com; 3 Department of Early Childhood Educare, Ching Kuo Institute of Management and Health, Jilong 20301, Taiwan

**Keywords:** enthusiasm for engagement, mental health literacy, moderation, online learning community, preschool teachers

## Abstract

*Background*: Most of the existing literature analyzes preschool teachers’ perceptions of information seeking and measures their satisfaction with online support for mental health issues. Seldom has this literature considered the influence of enthusiasm for or preference towards online engagement and social media in the development of preschool teachers’ mental health literacy. *Methods*: This study focused on preschool teachers’ attitudes towards the impact of an online learning community on mental health literacy and explored the moderation of enthusiasm for engagement on this relationship. A survey was conducted in Taiwan, and the researchers employed partial least squares to test the moderating effect. *Results*: The results indicate that enthusiasm for engagement has a negative moderating effect on the relationship between an online learning community and mental health literacy for preschool teachers. *Conclusions*: The moderating effect of enthusiasm for engagement in this relationship reminds us to consider the advantages and disadvantages of the employment of online learning communities for the improvement of mental health literacy and well-being. This study recommends cautiously integrating online learning communities and real-world communication into an appropriate and user-friendly interactive model to help preschool teachers promote their mental health literacy and well-being.

## 1. Introduction

The mental health conditions of preschool teachers play a critical role in their educare practices and professional development in early childhood education. Preschool teachers with better mental health literacy tend to support appropriate understanding, recognition, and beliefs about mental health [1]. With the development of social media, online learning communities provide preschool teachers with more opportunities to recognize mental health disorders and interact with community members in order to articulate empathic support and develop strategies for promoting well-being [2]. They can employ online learning communities to access mental health information and develop positive attitudes towards dealing with and discussing their mental health issues.

In Taiwan, the majority of preschool teachers are female and are educated to the level of a college degree. They have often been regarded as caregivers or babysitters, and so, less attention has been paid to their professional status as workers in early childhood education. In most cases, parents acknowledge the value of preschool teachers in the professional pedagogy of young children. However, preschool teachers often face undue pressure from parents due to parents having unreasonable expectations of preschool teachers and taking inappropriate pedagogical actions against them. As a result, they are unable to appropriately cope with their mental health problems; they cannot use the useful suggestions from mental health literacy as a way of reducing their mental and pedagogical stress.

Preschool teachers have an array of digital tools. They can access the internet and speak with online communities. Teachers can schedule online activities and communications to fit in with their other study needs and leisure activities, bettering their quality of life as a result. They can also use mobile apps and online instructional models to assist young children in their learning and improve their own professional and pedagogical skills.

Furthermore, most preschool teachers have the ability to use online learning communities and advance their mental health literacy in order to address their mental health problems or negative behaviors. However, some teachers are addicted to the internet and have expressed psychological problems. We found that some preschool teachers preferred real interactions and community support to improve their mental health conditions. Most of the existing literature analyzes preschool teachers’ perceptions of information seeking and measures their satisfaction with online support for mental health issues. The influence of enthusiasm for or preference towards online engagement and social media in the development of preschool teachers’ mental health literacy has seldom been considered. Therefore, this study explored preschool teachers’ attitudes towards the relationship between online learning communities and mental health literacy and clarifies the moderating effect of enthusiasm for engagement in this relationship.

### 1.1. The Relationship between Online Learning Communities and Mental Health Literacy

Online learning communities refer to spaces that users can access in order to learn specific branches of knowledge, improve their competencies, and advance in their professions. They are made possible by digital technology, and examples include social media channels such as Line and Facebook. Users employ different methods in order to search and gain access to these communities. They register as members online in order to gain important information for their working or life. These online learning communities are formed by professional partners, and they are useful for sharing knowledge or experiences. Through them, users spread critical viewpoints and thoughts that members then learn from as a way of improving their skills. Preschool teachers, for instance, use online learning communities to improve their knowledge and experience of mental health issues by sharing and discussing experiences related to mental health with others in these spaces [3].

Users can easily access and use online learning communities to acquire mental health knowledge and, thereby, make healthier choices and adopt more beneficial practices [4]. Most preschool teachers trust the friendly and professional discussions in online learning communities and use them to promote the development of mental well-being for themselves as individuals and in their wider communities.

Mental health literacy is defined as an individual’s knowledge and beliefs about their own and others’ mental health disorders, as well as self-help strategies for reducing mental disorders [5]. With the proliferation of digital technology and social media, mental health literacy can be accessed and learned through online learning communities to improve one’s health status and establish a better foundation for well-being [6]. When preschool teachers have improved mental health literacy, they exercise a healthier lifestyle and perform better both professionally and pedagogically. 

Preschool teachers employ online learning communities to seek professional assistance, to better their mental health, to resolve problems related to mental disorders, and to enhance online self-efficacy for the improvement of their mental health conditions. Based on these ideas, we propose the following hypothesis:

**Hypothesis** **1.** *Online learning communities positively influence mental health literacy.*

### 1.2. The Relationship between Enthusiasm for Engagement and Mental Health Literacy

Social engagement and enthusiasm for online communication play an important role in the development of adults’ mental health status [7]. Enthusiasm for engagement refers to the process of individuals spending more effort and time engaging in online activities [8]. As such, they see a virtual world based on online technology as being more valuable than real life. Individuals with high enthusiasm for engagement prefer online interactions to face-to-face ones. They perform a digital role in an online community. This is the way in which they share and exchange life experiences and emotions with others.

Enthusiasm for engagement indicates that more engagements with an online community help individuals lead more meaningful lives [9]. People who have enthusiasm for engagement are passionate about sharing their experiences with mental health through online interactions and are more positive about seeking health information and resolving mental health issues online.

Online engagement enables preschool teachers to develop a sense of social connectivity, helps to reduce negative effects and behaviors associated with mental health disorders, and helps to mitigate negative thoughts in the workplace. Enthusiasm for engagement fosters their emotional access to other teachers, increases their well-being, and provides teachers with a more confident understanding of mental health conditions. Preschool teachers engage enthusiastically in online learning communities to access health information and to develop and encourage optimistic attitudes towards mental health. By doing so, they can resolve their mental health problems and lead a meaningful and healthy life. Based on this, we propose the following hypothesis:

**Hypothesis** **2.** *Enthusiasm for engagement positively influences mental health literacy.*

### 1.3. The Moderating Role of Enthusiasm for Engagement

Online learning communities provide varied and user-friendly information for users to communicate with others about mental health issues. Preschool teachers show positive attitudes towards enthusiasm for engagement to employ social media and support their information-seeking behaviors and reduce mental health issues [10]. Compared with users who do not engage online with mental health resources, users with more enthusiastic perceptions often engage in online learning communities by actively posting and interacting with mental health content and strategies. Behavioral intentions and enthusiasm for engagement via social media influence users’ experiences of health and prompt them to develop their competence in mental health literacy.

**Figure 1:**
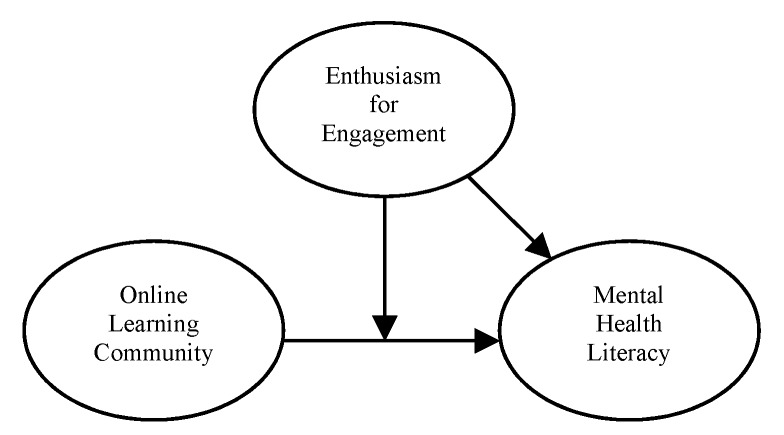
The theoretical model.

In fact, individuals with positive enthusiasm for engagement in online learning communities tend to develop better in relation to depression, anxiety, and mental well-being [11]. Enthusiasm for engagement helped preschool teachers actively access mental health information and increase their mental well-being. However, we found that some preschool teachers preferred face-to-face interaction over online interaction in relation to sharing and discussing mental health issues. The mechanism of enthusiasm for engagement in the relationship between online learning communities and mental health literacy should thus be considered and investigated. Therefore, we propose the following hypothesis and theoretical model as seen in Figure 1:

**Hypothesis** **3.** *Enthusiasm for engagement moderates the relationship between online learning communities and mental health literacy.*

## 2. Materials and Methods 

### 2.1. Sample Characteristics

We used an initial sample of 600 preschool teachers in Taiwan. After excluding blank responses or those with missing values, the valid sample size comprised 534 anonymous preschool teachers with a response rate of 89%. The percentages of male and female respondents were 2.81% and 97.19%, respectively. Most of the respondents were between 40 and 49 years of age (37.27%), whereas 29.03% were aged between 30 and 39 years. Most of the respondents had a university degree (64.23%) and served in a private preschool (60.49%). Almost half of the respondents (48.31%) had more than 11 years of experience of accessing online learning communities, and 33.25% of the respondents had between 5 and 10 years of experience.

### 2.2. Measurement Instrument

This study focused on preschool teachers’ attitudes towards the relationship between online learning communities and mental health literacy and the impact of that relationship. It also investigated the moderating role of enthusiasm for engagement in this relationship. A Chinese questionnaire, the “Preschool Teachers’ Mental Health Literacy Scales (PTMHL)”, was developed for this study. Based on the literature review and the theoretical hypotheses of this study, we developed the observed variables of the PTMHL and consulted with scholars and experts in the field of online technology and preschool education.

The PTMHL was comprised of three factors: “online learning community”, “mental health literacy”, and “enthusiasm for engagement”. The original survey instrument comprised 18 observed variables (six variables for each latent construct) and presented statements for which the respondents indicated their degree of agreement/disagreement on a 5-point Likert scale (1 = most strongly disagree and 5 = most strongly agree). A description of the three latent constructs is presented as follows:

1. Online learning community (OLC): assessing preschool teachers’ attitudes towards preschool teachers’ competence for using online learning communities to access knowledge and resources about mental health issues and interact with others to share their experiences and perceptions on this topic.
2. Mental health literacy (MHL): investigating the extent of preschool teachers’ perceptions of their mental health status and their capacity to develop appropriate mental literacy to resolve their possible mental illnesses and achieve mental health well-being.
3. Enthusiasm for engagement (EE): assessing preschool teachers’ attitudes towards online learning communities, their enthusiasm for and positive perceptions of social media, and their willingness to access online learning communities.

### 2.3. Data Analysis

The survey data were analyzed by using partial least squares and tested the interactive effect of the moderation [12,13]. We employed SmartPLS 3 (SmartPLS GmbH, Oenningstedt, Germany) [14] to analyze the raw data and to test the internal consistency reliability, convergent validity, and discriminant validity of the estimations in the measurement model. The factor loading of the different items was estimated by a confirmatory factor analysis and used 5000 bootstrap replications to examine statistical significance. We reported the Cronbach’s alpha, composite reliability (CR), average variance extracted (AVE), and correlation coefficient to test the reliability and validity of the latent constructs. In the analysis of the structural model, we assessed the estimations of standardized regression coefficients and the explanatory power of hypothetical paths. This study reported the goodness of fit (GoF) index, total effects, and their *t* statistics to evaluate the significance of the whole model by the bootstrap estimation. The moderating effect in the hypothesized relationship was tested on the statistical principle of partial least squares and examined the supporting results of the research hypotheses [15,16].

## 3. Results

### 3.1. Measurement Model

We used confirmatory factor analysis to evaluate the observed variables and latent constructs in the measurement model. According to the analysis of factor loadings and model fit indices per latent construct, a reflective variable only held when its loading was greater than 0.700 on the latent construct. The initial 18 observed variables were reduced to 15 for the PTMHL). The mean values of the retained 15 observed variables ranged from 3.530 to 4.116, and the standard deviations (SD) ranged from 0.602 to 0.874. The standardized factor loadings for each variable ranged from 0.779 to 0.938, as shown in Table 1. We used the bootstrapping method, based on 5000 samples, to test the level of significance of the standardized factor loadings. The results showed that the *t* statistics of the observed variables were all greater than 3.29, which indicated statistical significance (*p* < 0.001).

The Cronbach’s alpha, CR, and AVE values of each latent construct of the PTMHL ranged from 0.881 to 0.950 (which is higher than the accepted value of 0.800), from 0.913 to 0.962 (which is higher than the accepted value of 0.800), and from 0.678 to 0.835 (which is higher than the accepted value of 0.500), respectively, as shown in Table 2. The correlation of the latent constructs ranged from 0.391 to 0.595. The correlation coefficients between each latent construct were less than the respective square root of the AVE, which ranged from 0.824 to 0.914.

Table 3 shows the detailed analysis using the heterotrait–monotrait ratio of correlations (HTMT) technique. All the HTMT values were less than a cutoff value of 0.85 and ranged from 0.425 to 0.645. These measurements depict a reasonable degree of reliability, convergent validity, and discriminant validity of the latent constructs. The results suggest that the PTMHL measurement model had a high reliability of internal consistency.

### 3.2. Structural Model

Table 4 shows the results of the structural model and indicates the model quality. All statistics reported the appropriate level of the cutoff values, such as *R*^2^, Adj. *R*^2^, *f*^2^, *Q*^2^, VIF, and SRMR. According to the path coefficients and related statistics in the structural model of the PTMHL, the LC and EE constructs explain 37.2% of the proportion variance of the MHL construct, corresponding to a standardized regression coefficient of 0.528 and 0.147, respectively. All path coefficients were extremely statistically significant (*p* < 0.05) after performing a bootstrap with 5000 resamples, supporting both hypothesis 1 (H1) and hypothesis 2 (H2). The goodness of fit (GoF) index is 0.521, which is higher than the accepted value of 0.36 [17], and indicates that the structural model had reasonable quality and high explanatory power. The results show that preschool teachers had positive perceptions of employing online learning communities to access mental health information, and their enthusiasm for social media supported the online behaviors and communications that helped them deal with their mental health conditions.

### 3.3. The Moderating Effect of Enthusiasm for Engagement

We used SmartPLS 3 to convert the independent variable and moderating variable into standardized scores to calculate the interactional terms for moderating analysis. The study also employed the bootstrapping method to perform 5000 resamples to test the statistical significance of the moderating effect. According to the results, the path coefficient of the interaction effect terms of standardized indicator values before multiplication with the OLC and EE constructs on the MHL construct is −0.112 and the *t* value is 3.271. The 95% bias-corrected bootstrap confidence interval of the moderator’s effect is [−0,181, −0,048], and the confidence interval does not include zero. The results show that the moderating effect of enthusiasm for engagement is significant in this relationship.

This study demonstrates the negative moderating effect of enthusiasm for engagement on the relationship between online learning communities and mental health literacy. The explanatory power of the main effect is 0.372, the explanatory power of the moderating effect is 0.389, and the effect value of the moderation is 0.028, with a small moderating effect. These results support hypothesis 3 (H3). In summary, preschool teachers with lower inclinations towards participating in online learning communities and interacting with others online carried more positive attitudes towards accessing online communities to improve their mental health for their career development.

## 4. Discussion

This study explored preschool teachers’ attitudes towards the relationship between online learning communities, mental health literacy, and enthusiasm for engagement. The results show that enthusiasm for engagement plays a negatively moderating role in the relationship between online learning communities and mental health literacy, supporting hypotheses H1–H3. We found that online learning communities helped preschool teachers to develop strategies to access mental health information and to discuss their mental health problems or behavioral intentions with community members. They could share their mental experiences and employ the appropriate mental health strategies to address negative mental states, such as depression and anxiety.

Importantly, the study indicates that enthusiasm for engagement played a critical moderating role in the process of improving their mental health literacy via online communities. Preschool teachers with positive perceptions about enthusiasm for engagement showed little preference for the employment of online learning communities and social media to promote their mental health literacy. They preferred interacting with others via face-to-face communication to share their mental health experiences and seldom thought about how to resolve mental health issues via an online learning community. The online interactive model provided more virtual and multidimensional communicative opportunities but could not help them find warm and friendly emotional support to promote their mental well-being.

In Taiwan, preschool teachers spend a lot of time and effort engaging in online communities in order to access more information about their lives and to carry out further study. They engage enthusiastically with the internet and with online applications, which helps to generate positive attitudes. If they are more engaged in the virtual world, though, this means they are more likely to be alienated in the real world. When they engage in online communities, they experience fewer prosocial emotions. This makes it more difficult for them to deal with their mental health issues. They find themselves in less warm or friendly online environments, which can lead to depressed emotional states. Although online communities provide teachers with more useful information and knowledge to advance their literacy regarding mental health, they do not feel the benefits of this in their own mental health.

The moderating effect of enthusiasm for engagement in this relationship reminds us to consider the advantages and disadvantages of the employment of online learning communities for the improvement of mental health literacy and well-being. Uses of social media, such as Facebook, Line, WeChat, and QQ, may open up our perspectives about human beings and the world, but these digital tools can also have negative effects on our living and mental health status. Some of the respondents indicated an addiction to the internet, demonstrated little to no enthusiasm for online engagement, and easily displayed negative symptoms and behaviors associated with mental health problems while online.

The findings of this study suggest that we have to integrate online learning communities and real-world communication, cautiously, into an appropriate and user-friendly interactive model to help preschool teachers promote their mental health literacy and well-being. The practices of offline interaction through sincere dialogue can provide friendly and caring contexts, which support preschool teachers in improving their mental health literacy and status.

## 5. Conclusions

We explored preschool teachers’ attitudes towards the relationships of online learning communities, mental health literacy, and enthusiasm for engagement. The results supported H1, H2, and H3. They help us understand that enthusiasm for engagement negatively moderates the relationship between online learning communities and mental health literacy. Preschool teachers can employ online learning communities to learn about mental health, to share their mental health experiences, and to learn about appropriate mental health strategies.

The fact that enthusiasm for engagement has a moderating effect on this relationship reminds us to consider the advantages and disadvantages of the employment of online learning communities for the improvement of mental health literacy and well-being. It is recommended that future research adopt or adapt the PTMHL developed in this study. Furthermore, other latent factors could be taken into account, and other moderating variables and hypotheses could be considered. Specifically, this would help to test preschool teachers’ perceptions of online learning communities, mental health literacy, and enthusiasm for engagement.

This study focused on preschool teachers’ perceptions regarding mental literacy. It also focused on the ways in which those teachers access information about mental health via online communities. We were not able to address the teachers’ mental health or other related issues regarding psychotherapy or personal privacy. Future research could use appropriate observations or interviews in order to understand how preschool teachers evaluate their mental health and adopt useful and healthy strategies to improve their well-being.

## Figures and Tables

**Table 1:** The mean, standard deviations (SD), factor loading, and *t* value in the “Preschool Teachers’ Mental Health Literacy Scales” (PTMHL).

Item	Mean	SD	Factor Loading	*t* Value
OLC1	3.736	0.747	0.866	60.806 ***
OLC2	3.811	0.698	0.847	51.182 ***
OLC3	3.530	0.874	0.823	44.702 ***
OLC4	3.547	0.696	0.788	36.979 ***
OLC5	3.811	0.685	0.791	37.351 ***
MHL1	3.987	0.602	0.884	49.823 ***
MHL2	3.951	0.638	0.926	85.418 ***
MHL3	3.867	0.687	0.920	66.748 ***
MHL4	3.888	0.687	0.939	103.642 ***
MHL5	3.843	0.718	0.898	67.621 ***
EE1	4.116	0.641	0.824	41.833 ***
EE2	3.976	0.694	0.873	55.656 ***
EE3	3.762	0.773	0.812	38.610 ***
EE4	3.951	0.644	0.828	41.465 ***
EE5	3.863	0.650	0.778	32.291 ***

Note: *** = *p* < 0.001. OLC: online learning community; MHL: mental health literacy; EE: enthusiasm for engagement.

**Table 2:** The Cronbach’s alpha, composite reliability (CR), average variance extracted (AVE), and correlation matrix.

Factor	Cronbach’s Alpha	CR	AVE	OLC	MHL	EE
OLC	0.881	0.913	0.678	0.824		
MHL	0.881	0.913	0.678	0.463	0.824	
EE	0.950	0.962	0.835	0.595	0.391	0.914

Note: The square root of the AVE of two latent constructs is given on the diagonal, and the correlation coefficient is given on the below diagonal.

**Table 3:** Discriminant validity: heterotrait–monotrait ratio of correlations (HTMT).

Factor	OLC	MHL	EE
OLC			
MHL	0.645		
EE	0.525	0.425	

**Table 4:** The structural model results.

Factor	*R* ^2^	Adj. *R*^2^	*f* ^2^	*Q* ^2^	VIF	SRMR
OLC			0.348		1.273	
MHL	0.372	0.369		0.288		0.054
EE			0.027		1.273	
